# Similarity of plant functional traits and aggregation pattern in a subtropical forest

**DOI:** 10.1002/ece3.2973

**Published:** 2017-04-26

**Authors:** Bo Zhang, Xiaozhen Lu, Jiang Jiang, Donald L. DeAngelis, Zhiyuan Fu, Jinchi Zhang

**Affiliations:** ^1^ Key Laboratory of Soil and Water Conservation and Ecological Restoration in Jiangsu ProvinceCollaborative Innovation Center of Sustainable Forestry in Southern ChinaNanjing Forestry UniversityNanjingChina; ^2^ Department of BiologyUniversity of MiamiCoral GablesFLUSA; ^3^ Wetland and Aquatic Research CenterU. S. Geological SurveyGainesvilleFLUSA

**Keywords:** demographic traits, environmental filtering, functional traits, plant distribution, soil heterogeneity

## Abstract

The distribution of species and communities in relation to environmental heterogeneity is a central focus in ecology. Co‐occurrence of species with similar functional traits is an indication that communities are determined in part by environmental filters. However, few studies have been designed to test how functional traits are selectively filtered by environmental conditions at local scales. Exploring the relationship between soil characteristics and plant traits is a step toward understanding the filtering hypothesis in determining plant distribution at local scale. Toward this end, we mapped all individual trees (diameter >1 cm) in a one‐ha subtropical forest of China in 2007 and 2015. We measured topographic and detailed soil properties within the field site, as well as plant leaf functional traits and demographic rates of the seven most common tree species. A second one‐ha study plot was established in 2015, to test and validate the general patterns that were drawn from first plot. We found that variation in species distribution at local scale can be explained by soil heterogeneity and plant functional traits. (From first plot). (1) Species dominant in habitats with high soil ammonium nitrogen and total phosphorus tended to have high specific leaf area (SLA) and relative growth rate (RGR). (2) Species dominant in low‐fertility habitats tended to have high leaf dry matter content (LDMC), ratio of chlorophyll a and b (ratioab), and leaf thickness (LT). The hypothesis that functional traits are selected in part by environmental filters and determine plant distribution at local scale was confirmed by the data of the first plot and a second regional site showed similar species distribution patterns.

## 1 Introduction

The distribution of species and community assembly along environmental gradients is a central focus in ecology (Liu et al., 2011). Trait‐based approaches have been proposed to understand community assembly of traits selected by environmental conditions (Cingolani, Cabido, Gurvich, Renison, & Diaz, 2007; Wright et al., 2004; Zheng & Shangguan, 2007). Plant species differ in their success in heterogeneous environmental conditions (Cunningham, Summerhayes, & Westoby, 1999; Luzuriaga, Gonzalez, & Escudero, 2015; Westoby & Wright, 2006) due to their different physiological and life cycle traits (Ackerly & Cornwell, 2007; Kusumoto, Enoki, & Kubota, 2013; Meziane & Shipley, 1999; Shipley, 2010; Shipley, Vile, & Garnier, 2006).

Environmental filtering, which influences species occurrence in particular sites, is a key factor in community assembly and species distribution (McGill, Enquist, Weiher, & Westoby, 2006). Across regional or global scales, climate limits plant species distributions. For example, the ranges of boreal, temperate forest, and tropical forest are normally determined by temperature. Arid or semi‐arid grassland is usually distributed in low precipitation areas. Many species distribution models with climate envelope are developed to predict species distributions (Pearson & Dawson, 2003). While those large‐scale studies are important for understanding species distribution, local scale heterogeneity influences community assembly through interactions between abiotic and biotic factors. One component of the environmental filtering process at the local scale is edaphic heterogeneity (e.g., Baldeck et al., 2012; Barot, Gignoux, & Menaut, 1999; John et al., 2007; Oliveira‐Filho, Vilela, Carvalho, & Gavilanes, 1994; Reynolds, Mittelbach, Darcy‐Hall, Houseman, & Gross, 2007), which, in part, is generated by variations in topography (Burke et al., 1999; Homeier, Breckle, Gunter, Rollenbeck, & Leuschner, 2010; Lan, Hu, Cao, & Zhu, 2011; Newton, Peres, Desmouliere, & Watkinson, 2011; Pakeman, Lepš, Kleyer, Avorel, & Garnier, 2009; Silver, Scatena, Johnson, Siccama, & Sanchez, 1994), and which influences the spatial distribution of plant populations (Fayolle et al., 2012; Harpole & Tilman, 2007; Russo, Davies, King, & Tan, 2005). Such influence is more evident on smaller spatial scales (e.g.,<1 km^2^) (Mason et al., 2011), as dispersal is usually not a limitation on species distribution at small scales, so that the distribution of plant species reflects the influence of abiotic filters and biotic interactions, collectively referred to as the community assembly process (Weither & Keddy, 1999).

Focusing on the value of plant functional traits helps to understand the “fit” of organisms to their environment (Ackerly, 2003). If different species have similar traits, they should be expected to occupy the same range of environments; that is, their fundamental niches should be the same. This has been formalized in the idea of the functional niche in terms of the common traits of co‐occurring species (Rosenfeld, 2002). The co‐occurrence of species with similar traits is considered to be the result of environmental filters and species competition (Keddy, 1992; Cavender‐Bares, Ackerly, Baum, & Bazzaz, 2004; Cornwell et al. 2006). However, how the functional niche is governed by traits within the context of abiotic environmental gradients is not well‐known (Harper, 1980; Jager, Richardson, Bellingham, Clearwater, & Laughlin, 2015; Pulliam, 2000; Reich et al., 2003).

The objective of this study was to test the importance of soil characteristics acting as environmental filters on multidimensional traits, and thus to understand the community assembly and plant distribution at local scales. We hypothesize that environmental filtering generated by soil heterogeneity results in aggregations of species with similar traits. We mapped all individuals (diameter > 1 cm) in a one‐ha subtropical forestplot in Zhejiang Province, China, in 2007 and 2015. We measured topographic and soil properties within the field site, as well as plant leaf functional traits and demographic rates of individuals of the seven dominant tree species. Specifically, we asked the following: (1) Are species aggregated into distinct assemblages due to the environmental filtering in terms of the functional traits? (2) Which traits are most decisive in determining spatial distribution, and which physical properties do those traits interact with? (3) Are clumped patterns in species sharing similar functional traits consistent across sites?

## 2 Materials and methods

### 2.1 Study sites

The study area is located in the Fengyang Mountain National Natural Reserve, Zhejiang Province, southeastern China (119°06′‐119°15′E,27°46′‐27°58′N). This area was established as a national nature reserve in 1975, and the entire study area has been protected from anthropogenic disturbances (Guo, Meng, Zhang, & Chen, 2016; Guo, Chen, et al., 2016). The reserve is located in subtropical mixed deciduous/coniferous forest. Mean annual precipitation is approximately 2,400 mm, with highest rainfall occurring from May to August, lowest from November to December. Mean annual temperature is 12.3°C, and the average minimum temperature in January is 2.4°C.

The first 1‐ha (100 m*100 m) study plot (plot 1) was established and investigated during the summer of 2007 and then reinvestigated in the summers of 2015 (119°10′17.28′′E,27°52′38.49′′N). The plot has two slopes (low slope: the mean angle derived from the horizontal <20° and high slope: the mean angle derived from the horizontal >20°) and two aspects, west‐facing (the two right columns in Figure 2) and east‐facing (the two left columns in Figure 2), with a valley down in the middle (the middle column in Figure 2). Topographic variations in slope are likely to be a source of soil heterogeneity at local scales, while differences in other factors, such as light and temperature, should be small. However, considering that such topographic variations might lead to variations in soil condition, we divided our plot into five topographic zones: low slope on west‐facing (WL), high slope on west‐facing (WH), valley in the middle (VV), low slope on east‐facing (EL), and high slope on east‐facing (EH). The elevation range of the reserve is between 600 and 1,929 m (Guo, Chen, et al., 2016; Guo, Meng, et al., 2016). We divided the plot evenly into 25 subplots, each 20 m*20 m in area. The topographic map of the plot is shown in Fig. S1.

A second 1‐ha (100 m*100 m) study plot (plot 2) was randomly selected within the same study area (119°10′3.58′′E,27°54′15.48′′N), established and investigated during the summer of 2015, to test and validate the general patterns that were drawn from first plot. (The reason that we selected the second plot at same site is to make sure both plots have same dominated species. However, we did not specify topography when choosing the plot, to test whether the pattern generated from plot 1 could predict other plots with similar species.) All the trees in both plots 1 and 2 with diameters at breast height (DBH) greater than 1 cm were labeled with aluminum alloy tags. We recorded species name, DBH, height, and coordinates for each tree.

### 2.2 Study species

Three canopy (over story tree) species, *Cyclobalanopsis stewardiana*(CYST), *Cyclobalanopsis multinervis* (CYMU), and *Schima superba* (SCSU), and four understory tree species, *Rhododendron simiarum* Hance (RHSH), *Camellia cuspidate* (CACU), *Rhododendron simsii* Planch (RHSP), and *Rhododendron latoucheae* (RHLA), were selected in our analysis, of a total of 15 woody plant species in plot 1. These seven species are important components of subtropical forest in southeastern China and they are the dominant species, accounting for 70% of total basal area in plot 1. These species represent a range of life‐history traits, including variation along the spectrum from shade tolerant to shade intolerant.

### 2.3 Quantification of site properties in plot 1

To characterize spatial heterogeneity in plot 1, topographic and soil data were measured at each 20*20 m subplot. Topographic attributes were measured by TOPCON electro‐optic Total Station (Topcon Corporation, Tokyo, Japan), including mean elevation, slope, and aspect. For each of the 25 400‐m^2^ subplots, the mean elevation was defined as the average over the elevations of its four corners, and the slope was the mean angle derived by averaging the slopes of the four triangular planes formed by connecting the four possible combinations of three of its adjacent corners. Aspect refers to the horizontal direction toward which the mountain slope faces. Canopy density (Canopy) and the amount of canopy cover (0% means totally open and 100% means total canopy closure) were visually estimated from tree density.

Soil sample (0–10 cm) extractions involved analysis of three replicate soil cores (two in the corners and one in the center) in each of the 25 subplots in plot 1 to determine variations in soil pH, organic matter content (OC %), total nitrogen (TN g/kg), total phosphorus (TP g/kg), ammonium nitrogen (NH_4_ g/kg), available phosphorus (AP mg/kg), and available potassium (AK g/kg), calcium (Ca g/kg), and magnesium (Mg g/kg).

The soil pH was measured in a 1:5 soil–water leachate, by a standard pH meter (Sartorius, PB‐10). Percent organic carbon (OC%) was determined by the K_2_Cr_2_O_7_ titration method. Total nitrogen (TN) was estimated by the Kjeldahl nitrogen determination method. The C/N ratio of soil organic C and soil total N could be determined from these data. TP was detected by sulfuric acid—hydrochloric acid heating digestion method. The NH_4_ was assayed at 645 nm by indophenol blue colorimetry, and the weight ratio of media to extractant (2 mol/L KCl) was 1:5. The AP was estimated using the double acid leaching molybdenum antimony colorimetric method. The AK was estimated using NH_4_Ac extraction flame photometry. The method for determining soil Ca and Mg was atomic absorption spectrometry (AAS). The main properties measured in each subplot are listed in Table S1.

### 2.4 Physiological function traits data in plot 1

Physiological function traits related to plant establishment and persistence were measured for the seven species. Each species was sampled in each subplot of plot 1. These included both leaf functional traits (leaf size, specific leaf area (SLA), leaf dry matter content (LDMC), ratio of chlorophyll a and b (ratioab) and leaf thickness (LT)), and demographic traits (mean height, relative growth rate (RGR), survivorship, and reproduction rate).

Leaf size (surface area in cm^2^), specific leaf area (SLA) (leaf area/leaf dry mass *10 in m^2^/kg), leaf dry matter content (LDMC) (leaf dry mass/leaf fresh mass), ratio of chlorophyll a and b (ratioab), and leaf thickness (LT) were calculated from field measurements of sun‐exposed fully expanded fresh young and undamaged leaves. Six trees of comparable size per species per subplot and two leaves per tree (different branches) were randomly sampled. Leaves were collected and transported in plastic bags wrapped with wet gauze and taken to the laboratory for immediate measurement. Measurements were performed on entire leaves (including petioles). Leaf area was calculated by Portable Leaf Area Meter. For measuring dry masses, leaves were oven dried for 48 h at 75°C. A gram of fresh leaves of each species was preserved to determine the chlorophyll before they were dried. The chlorophyll was extracted with acetone and ethyl ketone (1:1 by volume) leaching solution. After filtering, the filtrate was measured by spectrophotometer. Mean relative growth rate (RGR) was calculated using the following equation:

RGR=(DBH2015−DBH2007)/(DBH2007),

where DBH2007 and DBH2015 are the DBHs in years 2007 and 2015, respectively.

Mean survivorship was calculated using the following equation:

Survivorship=(n2007−n2015)/(n2007),

where n2007 and n2015 are the number of stems of each species in each subplot in 2007 and 2015, respectively, and only stems that existed in 2007 were counted.

Population reproduction rate was calculated using the following equation:

Reproduction=nnew2015/n2007,

where nnew 2015 is the number of new stems in 2015 and n2007 is the number of stems in 2007 of each species in each subplot. Dead stems in 2015 were excluded.

We calculated abundance‐weighted mean functional traits values for each subplot in plot 1, which is the mean trait of each species multiplied by its relative abundance in each subplot.

Note that topographic, soil, and functional data were not available for plot 2, due to limitation by budget and human labor.

### 2.5 Statistical analysis of data in plot 1

We used Ripley's *K*‐function and the distribution function of nearest‐neighbor distances to quantify the spatial patterns (aggregation/regularity). We confronted our data with the null model of complete spatial randomness to quantify departures from the null model. The *K*(*r*) function is defined as the expected number of dots within distance *r* from a randomly chosen dot. Under complete spatial randomness, $K\left(r\right)=\pi r^{2}$. The *L*(*r*) is defined as $\sqrt{\frac{K(r)}{\pi }}-r$ and under complete spatial randomness the expected value of *L*(*r*) is zero. *L*(*r*) was calculated on different scales (*r*). The 95% confidence envelopes of the *L*(*r*) functions were estimated from 500 simulations using a random arrangement of dot positions and random translations. When the observed *L*(*r*) values were larger or smaller than the envelopes of the expected *L*(*r*) under the null hypotheses, the spatial pattern of the points, was either aggregated (clumped) or regularly distributed, respectively, at neighborhood distance of *r*. Considering that plot 1 consisted of two halves, one on an west‐facing slope and the other on an east‐facing slope and that species distributions could change dramatically on the different aspects, we divided the data into two 40 * 100 m plots representing the two aspects, to calculate the Ripley's *K*‐function. Furthermore, we calculated *L*(*r*) of the seven species in plot 2 as well, to confirm our hypothesis that species sharing similar functional traits has similar spatial distribution across plot sites. Given that the second plot does not have as large a variation in topography as in the first plot, we decided to calculate the *L*(*r*) of each species within the whole plot.

To determine the correlation of the distributions of each pair of species, we conducted bivariate L‐function analysis with the coordinates of each pair of species. This bivariate pattern analysis quantified how one species was distributed relative to other species to examine whether the two species occur on average more or less frequently as near neighbors than expected; that is, if they were positively/negatively correlated. The bivariate *K*‐function *L*(*r*) is defined as the expected number of one type of species (e.g., CYMU) found within a given distance *r* of another species (e.g., CYST). The 95% confidence envelopes of the *L*(*r*) functions were calculated from 500 simulations of the null model of complete spatial randomness, which assumed independence between two species. *L*(*r*) values larger than the confidence envelope indicated that the pattern of species 1 more closely followed the pattern of species 2 than expected under independence (i.e., there was positive correlation). Similarly, *L*(*r*) values smaller than the confidence envelope indicated on average a significantly different spatial pattern than that of species 1 within a given distance *r* of the pattern of species 2 (negative correlation).

Field surveys produced an environmental data matrix of the 25 sample subplots (each representing an area of 400 m^2^) and the corresponding physiological function traits of seven tree species within each subplot. Canonical correspondence analysis (CCA) tests were performed with the Vegan Package in R (Oksanen et al., 2016). We first performed CCA to examine association between abundance (number of trees of each species per subplot) and environmental factors (soil and topographic properties), via weighted averaging and multivariate regression techniques. We did this using the variance of each soil and topographic property in the species distribution. We then performed CCA to examine the association between the abundance and the abundance‐weighted mean plot traits. We performed the CCA by forward selecting the independent variables to evaluate their significance levels. Each variable was tested at the 5% confidence level with 1,000 random permutations.

We used one‐way ANOVA and Tukey's test to test for significant differences of soil chemical properties among subplots.

## 3 Results

### 3.1 The impact of topography on soil chemical properties

**Figure 1:**
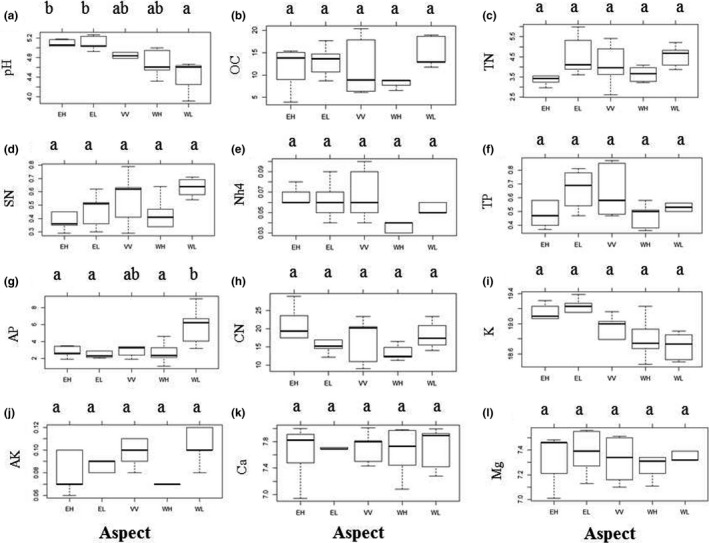
The impact of topography on soil chemical properties. Only significant environmental factors are shown. These are as follows: (a) pH: soil acidity; (b) OC: organic matter content; (c) TN: total nitrogen; (d) SN: soluble nitrogen; (e) NH_4_: ammonium nitrogen; (f) TP: total phosphorous; (g) AP: available phosphorus; (h) CN: ratio of carbon to nitrogen; (i) K: potassium; (j) AK: available potassium; (k) Ca: calcium; and (l) Mg: magnesium. The aspect was divided into five groups, as low slope on west‐facing (WL), high slope on west‐facing (WH), valley in the middle (VV), low slope on east‐facing (EL), and high slope on east‐facing (EH). The lower case letter above indicated significant different between aspects

Considering that there is a potential importance of topography on soil chemical properties, which could be related to the distributions of species traits that we found, we first analyzed the impact of topography on soil chemical properties. We found there is a significant impact of topography on soil pH and available phosphorus (AP). West‐facing with low slope (WL) had a significantly lower soil pH and higher AP than other topographies. However, we did not find significant differences in other soil properties among the five topographies (Figure 1).

### 3.2 Plant distributions differ among species and some groups of species are strongly aggregated in plot 1

**Figure 2:**
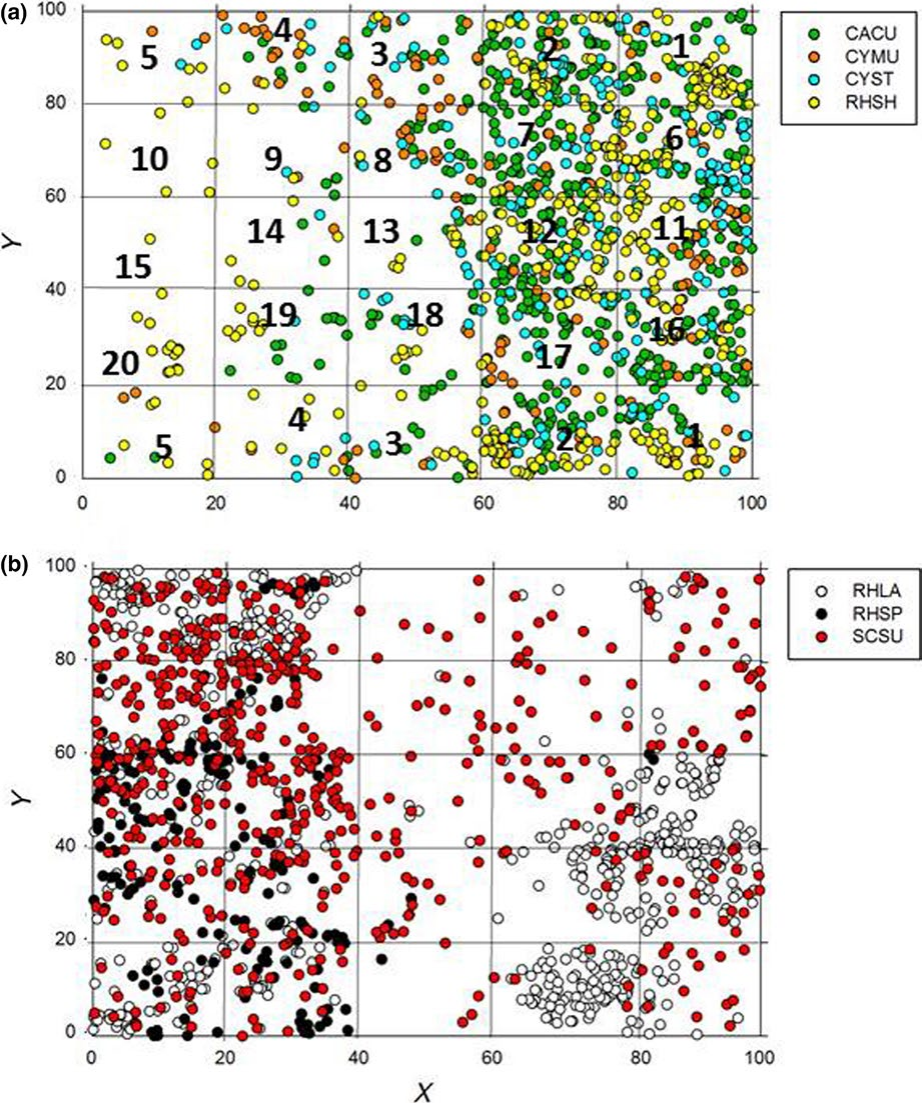
The spatial distributions of stems of each species in the study plot 1. (a) represents group 1 and (b) represent group 2. The study plot is 1 ha, and it is divided into 25 subplots (20 m*20 m). Light blue dots represent *Cyclobalanopsis stewardiana* (CYST); orange dots represent *Cyclobalanopsis multinervis* (CYMU); green dots represent *Camellia cuspidate* (CACU); yellow dots represent *Rhododendron simiarum* Hance (RHSH); red dots represent *Schima superba* (SCSU); empty (or white) dots represent *Rhododendron latoucheae* (RHLA); and black dots represent *Rhododendron simsii Planch* (RHSP). Numbers represents the ID of the subplots

The seven selected species showed different spatial stem distribution patterns among the 25 subplots of plot 1, but some groups of species had similar spatial patterns (Figure 2). CYST, CYMU, CACU, and RHSH mostly dominated in the subplots located on the right side (west‐facing) in Figure 2a (light blue, orange, green, and yellow dots; mainly in subplot numbers 1, 2, 6, 7, 11, 12, 16, 17, 21, and 22); in contrast, SCSU and RHLA showed similar spatially aggregated patterns that were widely located in the top left, bottom left, and bottom right subplots in Figure 2b (empty and red dots; mainly subplot numbers 4, 5, 9, 10, 14, 15, 19, 20, 24, and 25), whereas RHSP was only located in the left‐hand subplots, especially the top left (Figure 2b black dots; mainly subplot numbers 19, 20, 24, and 25). There was some dispersion of RHLA and SCSU to the subplots on the right side of Figure 2, but less dispersion of CYMU, CYST, CACU, and RHSH to the left side.

**Figure 3:**
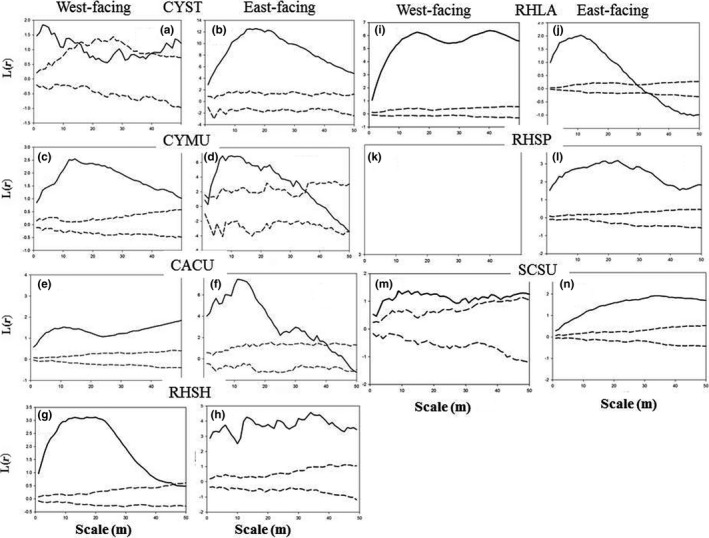
*L*(*r*) values of seven species. Solid lines indicate the univariate *L*(*r*) values of Ripley's *K*‐function; dashed lines indicated the upper and lower limits of the 95% simulation envelope of the *L*(*r*) functions. Lines above the upper envelope indicate clumped distribution, lines between the envelopes indicate random distribution, and lines below the lower envelope indicate regular distribution. (a, c, e, g, i, k) and (m) were from west‐facing aspect and (b, d, f, h, j, l) and (n) were from east‐facing aspect. (k) was empty, meaning the analysis of RHSP was unavailable, due to the stem numbers of RHSP on the west‐facing aspect was too small (only three stems were existed)

The univariate L‐function lines of the four species (CYST, CYMU, CACU, and RHSH) that mostly dominated on the west‐facing aspect of the plot showed similar aggregated distributions (the solid lines were above the upper envelope) at all scales (0–40 m) (Figure 3a,c,e,g). In contrast, those four species that had random distributions (the solid lines were between envelopes) at larger scales (>30 m) on the east‐facing aspect, except RHSH, which had a clumped distribution (Figure 3b,d,f,h). Compared to those four species, SCSU and RHSP kept clumped distributions at all the scales on the east‐facing aspect (Figure 3m,o), whereas SCSU had a very weakly clumped distribution (the solid line was close to upper envelope) on west‐facing aspect (Figure 3n). Interestingly, RHLA kept clumped distribution at all scales on west‐facing aspect and small scales (0–30 m) on east‐facing aspect and then change to a regular pattern at larger scales (solid line was lower than the lower envelope) (Figure 3j,k). We did not calculate the *L*(*r*) of RHSP on west‐facing aspect, due to there being only three stems, which did not qualify for statistical analysis.

**Figure 4:**
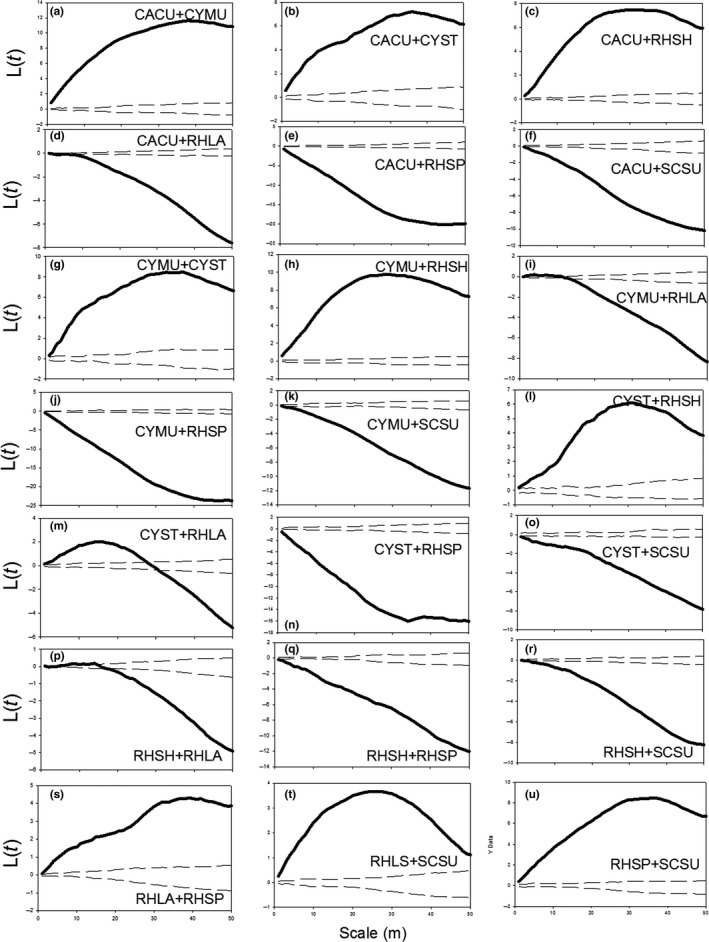
*L*(*r*) values of the correlation between all pairs among the seven species. Solid lines indicate the bivariate *L*(*r*) values of Ripley's *K*‐function; dashed lines indicated the upper and lower limits of the 95% simulation envelope of the *L*(*r*) functions. Lines above the upper envelope indicated positive correlations and lines below the lower envelope indicate negative correlations, between (a) CACU and CYMU; (b) CACU and CYST; (c) CACU and RHSH; (d) CACU and RHLA; (e) CACU and RHSP; (f) CACU and SCSU; (g) CYMU and RHLA; (h) CYMU and RHSP; (i) CYMU and SCSU; (j) CYMU and RHSP; (k) CYMU and SCSU; (l) CYST and RHSH; (m) CYST and RHLA; (n) CYST and RHSP; (o) CYST and SCSU; (p) RHSH and RHLA; (q) RHSH and RHSP; (r) RHSH and SCSU; (s) RHLA and RHSP; (t) RHLA and SCSU; and (u) RHSP and SCSU

The bivariate L‐function lines in Figure 4 show that there were positive correlations (the solid lines were above the upper envelope) between the species pairs that indicated similar patterns in Figure 2, whereas the correlations were negative (the solid lines were below the upper envelope) between the species that were conspicuously separate in Figure 2. Specifically, CACU had positive relationships with CYMU, CYST, and RHSH and negative relationships with RHLA, RHSP, and SCSU (Figure 4a–f); similar to CYMU, CYST, and RHSH, which had positive correlations with each other and negative correlations with RHLA, RHSP, and SCSU (Figure 4g–o); In contrast, RHLA, RHSP, and SCSU had positive correlations with each other (Figure 4s–u). Hence, the species fell into two distinct groups, and these two groups overlapped only slightly.

### 3.3 Selection of abundance‐weighted traits in different soil conditions

**Figure 5:**
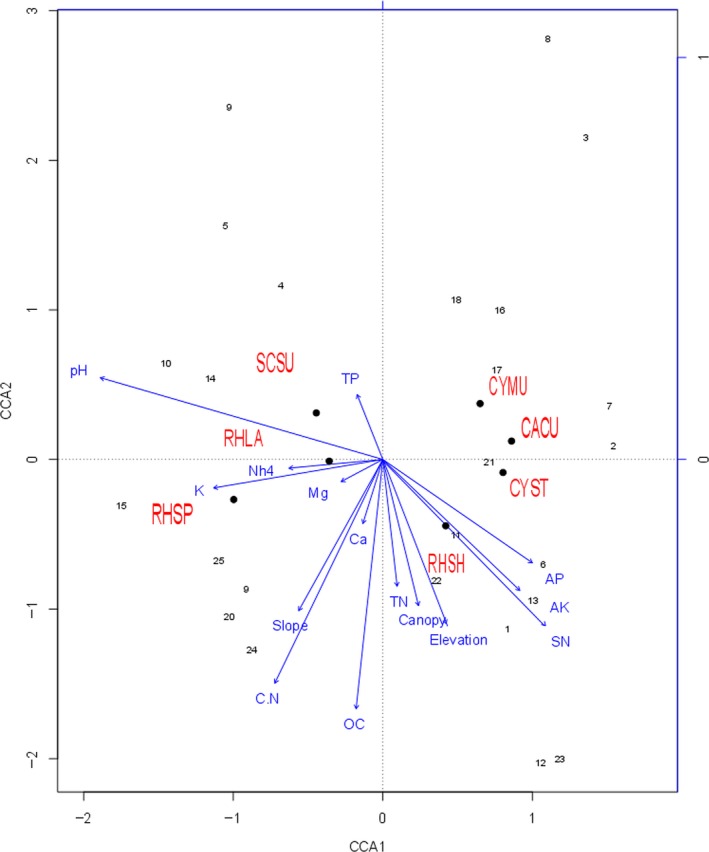
Canonical correspondence analysis (CCA) diagram displays the relation of the density of the seven species and selected soil factors. Only significant environmental factors are shown. These are as follows: pH: soil acidity, OC: organic matter content, TN: total nitrogen, SN: soluble nitrogen, NH_4_: ammonium nitrogen, TP: total phosphorous, AP: available phosphorus, C:N: ratio of carbon to nitrogen, K: potassium, AK: available potassium, Ca: calcium and Mg: magnesium, slope: the mean angular deviation from the horizontal, elevation: the average of the elevations of four corners of subplot, Canopy: the degree of canopy cover (0% means totally open and 100% means total canopy closure) was visually estimated from tree

The similarly aggregated patterns, such as CYST, CYMU, and CACU, being mostly dominant in the subplots that were located on the whole right half of the region in Figure 2a, appear to be explainable by the correlation of species’ densities and different soil nutrients shown in CCA analysis (Figure 5) as following:

1. CYMU, CYST, and CACU dominated in habitats (these are typically subplots 2, 7, 16, 17, and 18) with low nutrient level, that is ammonium nitrogen (NH_4_) lower than 0.09 g/kg, potassium (K) lower than 18.9 g/kg, and organic carbon content (OC) lower than 17.84%;
2. RSH dominated in the rich soil conditions with high available phosphorus (AP > 1.08 mg/kg), available potassium (AK > 0.06 g/kg), soluble nitrogen (SN > 0.29 g/kg), total nitrogen (TN > 2.61 g/kg), the degree to which the canopy is closed (Canopy) and elevation(typically subplots 1, 6, 13, and 22);
3. RHSP mainly occupied rich soil plots that had higher ammonium nitrogen(NH_4_), potassium (K), organic Carbon (OC), and ratio of carbon to nitrogen (C:N) (typically subplots 9, 15, 20, 24, and 25);
4. SCSU and RHLA dominated in the subplots (4, 5, 9, 10, and 14) that had higher pH and total phosphorous (TP).

The first two axes of CCA account for 74.66% of the variation of plant abundance among subplots in plot 1(Figure 5).

**Figure 6:**
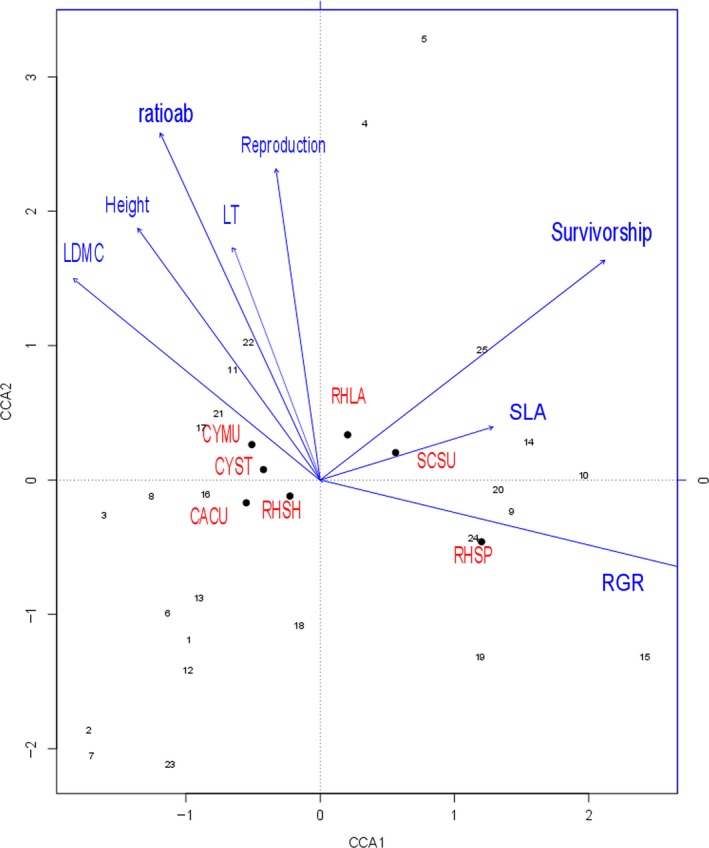
Canonical correspondence analysis (CCA) diagram displays the relation of the density of the seven species and abundance‐weighted mean plot traits. The specific leaf area (SLA), leaf dry matter content (LDMC), leaf thickness (LT), the ratio of chlorophyll a and b (ratioab), height, relative growth rate (RGR), survivorship and reproduction. Numbers represents the ID of the subplots in plot 1

We found that some particular soil conditions were more closely associated with plant species that shared certain similar traits, than with other species. For example, the subplots (11, 17, 21, and 22), which had low nutrient levels, were dominated by species that had higher leaf dry matter content (LDMC), leaf thickness (LT), ratio of chlorophyll a and b (ratioab), height, and reproduction rate. The subplots (9, 15, 19, 20, and 24), which had higher ammonium nitrogen (NH4), potassium (K), and organic carbon (OC), were dominated by fast‐growing species (high RGR). The subplots (4, 5, 14, and 25), which had higher pH, were associated with species that had higher specific leaf area (SLA) and survivorship. The first two axes of CCA accounted for 71.78% of the variation of abundance‐weighted mean subplot traits among plots (Figure 6). Different functional trait values under different subplots probably result from the selective pressure of soil nutrients.

### 3.4 Plant aggregation with similar functional traits is validated in plot 2

**Figure 7:**
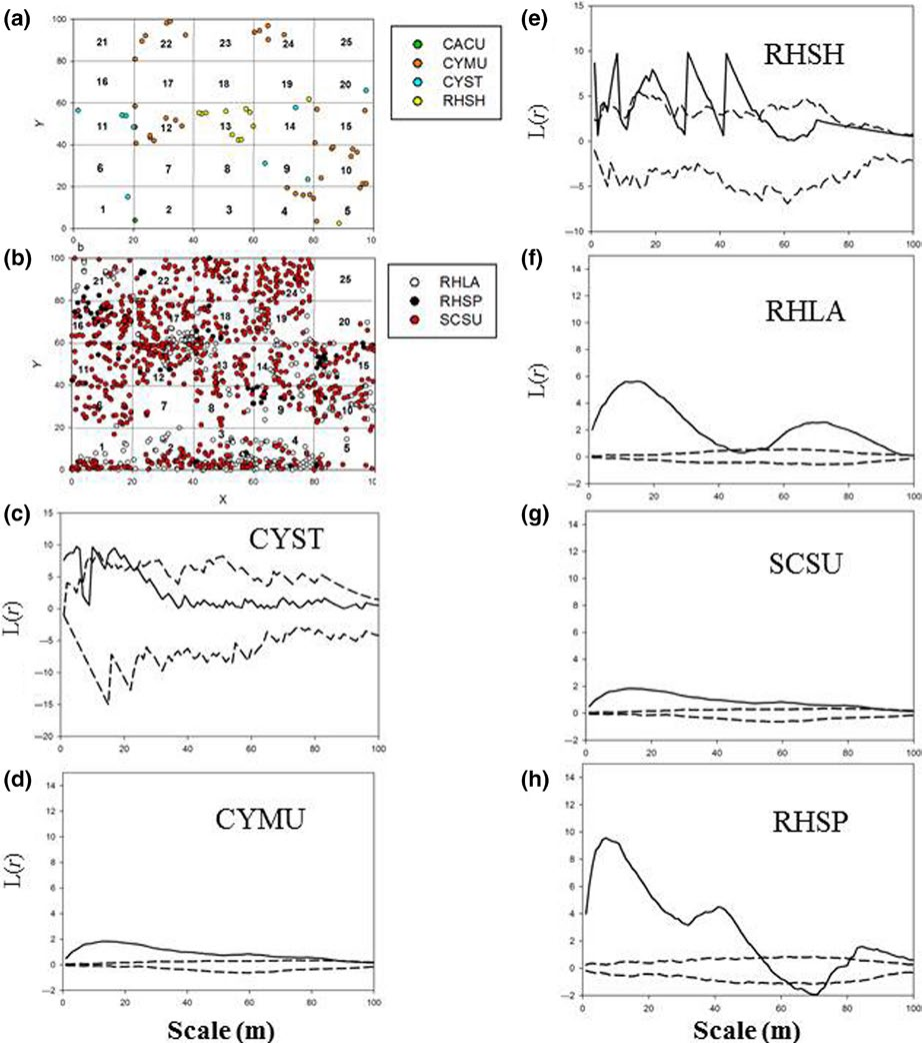
(a, b) The stem distribution patterns of the selective seven species in the study plot 2. The study plot is 1 ha, and it is divided into 25 subplots (20 m*20 m). Light blue dots represent *Cyclobalanopsis stewardiana* (CYST); orange dots represent *Cyclobalanopsis multinervis* (CYMU); green dots represent *Camellia cuspidate* (CACU); yellow dots represent *Rhododendron simiarum* Hance (RHSH); red dots represent *Schima superba* (SCSU); empty dots represent *Rhododendron latoucheae* (RHLA); and black dots represent *Rhododendron simsii Planch* (RHSP). Numbers represents the ID of the subplots. (c–h) showed *L*(*r*) values of seven species; solid lines indicate the univariate *L*(*r*) values of Ripley's *K*‐function; dashed lines indicate the upper and lower limits of the 95% simulation envelope of the *L*(*r*) functions. Lines above the upper envelope indicate clumped distribution, lines between the envelopes indicate random distribution, and lines below the lower envelope indicate regular distribution

The stem distribution patterns of the seven selected species were aggregated in plot 2 in a similar fashion (Figure 7) to what they were in plot 1 (Figure 2), although there are some striking differences in numbers. There were very few stems of CYMU, CYST, CACU, and RHSH in plot 2 (Figure 7a, light blue, orange, green, and yellow dots), whereas RHLA, RHSP, and SCSU were much more common and aggregated together in plot 2 (Figure 7b, empty, black, and red dots). Similar to the stem distribution patterns, CYST and RHSH were randomly distributed at all scales (0–100 m) (Figure 7c,e), CYMU had a weakly clumped distribution (the solid line was closer to the upper envelops) at small scales (0–60 m) and then ended with a random distribution at larger scale (Figure 7d). RHLA, SCSU, and RHSP had a clumped distribution on all scales (Figure 7f–h), except that the distribution of RHSP changed from random to regular to random at medium scales (60–80 m). Overall, our observations in plot 2 indicated that RHLA, RHSP, and SCSU, which had similar functional traits (such as SLA, RGR, and survivorship), and were aggregated together in plot 1, were also aggregated together in plot 2. Furthermore, the group RHLA, RHSP, and SCSU overlapped little with group CYMU, CYST, CACU, and RHSH in both plot 1 and plot 2.

## 4 Discussion

Our study showed that the seven dominant species formed two groups that were distinct in their spatial distributions. The traits of the species within each group were similar, and the two groups were distributed on sites that differed in their soil nutrient characteristics. The findings in plot 1 and 2 were consistent, in that the two groups comprising species with similar functional traits (located in plot 1) were spatially distinct, as shown from the Ripley's *K*‐function analysis.

Our results suggest that topographic variation might lead to differences in soil nutrient content, as observed in our findings that available phosphorus (AP) was higher at lower slopes of west‐facing aspect, which had lower soil pH (Xu et al., 2015). Such different environmental conditions may impose different selection forces on plants and drive traits to a certain degree of spatial divergence. Consequently, soil nutrient heterogeneity could explain the spatial variation of plant stem densities in our subtropical forest plots, due to the fact that differences of community composition are likely to be selected based on the niche axis of the functional traits. Our study did not find difference of soil moisture among the subplots. However, soil moisture could influence plant distribution through the trait of water use efficiency (Cornwell & Ackerly, 2009; Reich et al., 2003) and also has a strong relationship with tree mortality rate (Rigling & Bigler, 2013). It is unclear whether the relationship between soil moisture and plant distribution still holds in our study, but the spatial variation in soil moisture was smaller than the variation in soil nutrients. We cannot exclude the possibility that the causality has worked in the other direction as well; that is, that plants have affected the soil characteristics. Plants possess a multitude of traits that can lead to strong niche construction (i.e., plant‐induced changes in the soil) (Schweitzer et al., 2014), so that plants living in different environmental conditions (plant–soil linkages and feedbacks) could further change the local soil conditions. But the simplest explanation is that initial differences in soil characteristics existed that favored different plant assemblages.

Our results are consistent with other findings that most species show a relatively high stem clumping at the scale of these 20 × 20 m subplots (see references in Hara, Hirata, Fujihara, & Oono, 1996; Chang et al., 2012). In a study of a subtropical montane rainforest in the Fushan Forest Dynamics plot in northeastern Taiwan, Su, Hsieh, Chang‐Yang, Lu, and Guan (2010) found that nearly 30% of the variation in tree species composition was attributable to small‐scale topographic features. From an evolutionary perspective, it is of considerable importance to determine whether the force of natural selection is strong enough to enable different species to adapt and be more competitive on soils of slightly different chemical composition (Gartlan, Newbery, Thomas, & Waterman, 1986).

Our CCA analysis showed that different forms of a given nutrient were related to different functional traits, for example, soluble nitrogen, or nitrate associated with RHSH and ammonium nitrogen associated with RHSP. The abundance‐weighted functional trait values of a species can be hypothesized to influence its niche position and breadth along gradients (Violle & Jiang, 2009). This study is consistent with the idea that if a tree species is physiologically better adapted to certain soil conditions than its neighbors, it will grow at a faster rate and have a higher chance of domination in the local system. We found the group of species that was most aggregated on high ammonium nitrogen, potassium, and organic carbon plots had significantly higher RGR than in other plots, which supports the concept that fast‐growing species are better adapted to rich environments, while slow‐growing ones succeed better in low nutrient content habitats (Poorter & Bergkotte, 1992; Van‐Arendonk & Poorter, 1994).

Our results on the relative growth rate (RGR) are in agreement with Cunningham et al. (1999), Meziane and Shipley (1999), that plants such as SCSU and RHSP,with higher SLA (leaf area per unit biomass), tend to have greater potential relative growth rates (Garnier, 1992; Cunningham et al.,^1999^; Nathan and Muller‐Landau 2000). Also, high‐SLA species are opportunists that will colonize disturbances such as roadsides, canopy gaps, and small landslips (Garnier et al., 2001, 2004; Rosenfeld, 2002), which is consistent with our findings that high‐SLA species were located in the low canopy‐closedness subplots in which the canopy was not closed. Furthermore, species with higher SLA dominated in the plots with high soil pH and high total phosphorus, which agreed with Paoli (2006) that SLA increased significantly with higher soil nutrient.

Another group of species that had higher leaf dry matter content (LDMC), leaf thickness (LT), and ratio of chlorophyll a and b (ratioab) dominated in the plots that had low nutrient level. Because such species have thicker laminas, veins that protrude more, higher tissue density, or combinations of these (Shipley 1995; Niinements, 1999; Pyankov, Kondratchuk, & Shipley, 1999), they tend to achieve longer leaf lifespan in a variety of habitats (Reich, Walters, & Ellsworth, 1997; Ryser & Urbas, 2000; Wright & Westoby, 2002). Such species usually have lower specific leaf area (SLA) because plants from low‐fertility ecosystems have lower inherent growth rates, lower expected light capture per gram of dry mass invested in leaves, and higher carbon investment in secondary compounds allocated to storage and defense (Cunningham et al., 1999). These limits favor adaptation to low nutrient conditions (Grime et al., 1997; Reich et al., 1999). Therefore, high‐LDMC, LT, and ratioab species had large proportions of individuals in habitats where either low soil moisture, nutrient limitation, or both together strongly hamper growth. These subplots supported evergreen shrubs and small trees, which show the highest LDMC values (Porter et al. 2009).

We have neglected effects of seed dispersal here. Seed dispersal is commonly believed to influence community structure (Levine & Murrell, 2003) and may have some influence in our plots. But our hypothesis is that soil conditions are the underlying cause of spatial patterns and localized dispersal only reinforces these distributions (Ellner & Shmida, 1984). Another limitation of this study is that we did not test phylogenetic aspects of plants; that is, we did not test the effects of relatedness of the Rhododendron species. However, given that Rhododendron species do show different spatial patterns, the inclusion of the phylogenetic aspect might actually strengthen the point of functional differentiation due to the differences in the distribution of RHSH from that of both RHLA and RHSP. The final limitation of this study is that, although our study site represents a typical subtropical secondary forest in China, the number of species (seven species captures 70% of local stem density) is relatively low compared to other native forests in other regions of the world. We suggest further studies should be performed in high species richness regions such as tropical forest to test the hypothesis of coupled plant functional traits and species distribution.

## 5 Conclusion

In summary, our study validates the use of correlation between environmental factors and functional traits to better understand the underlying causes for different plant distributions along gradients in different environmental conditions (Lebrija‐Trejos, Perez‐Garcia, Meave, Bongers, & Poorter, 2010; Swenson & Enquist, 2007). We found that the widespread correspondence between phenotypic variation and environmental conditions reflects the functional traits (Sultan & Bazzaz, 1993). The differences of soil properties are hypothesized to influence functional traits of plant species that dominate particular sites. Plant attributes are fairly consistently associated with certain environmental conditions and are the consequence of the filtering effect of climatic, disturbance, and biotic conditions (Diaz, Cabido, & Casanoves, 1998).

## Conflict of Interest

None declared.
