# Supplementary material for: Similarity of plant functional traits and aggregation pattern in a subtropical forest
Source: Ecol Evol. 2017 Apr 26;7(12):4086–98. doi: 10.1002/ece3.2973 (PMC5478052; doi:10.1002/ece3.2973)
Supplement: Supplementary file 1 [file ECE3-7-4086-s001.docx]

Fig. S1 The topographic environment of the 25 sub-plots in the 1-ha field site (plot 1) on Fengyang Mountain National Natural Reserve, Zhejiang Province, China. A three-dimensional visualization of plants, the scope of fixed sample plot and superposition of the terrain are shown.

**Fig. S1**


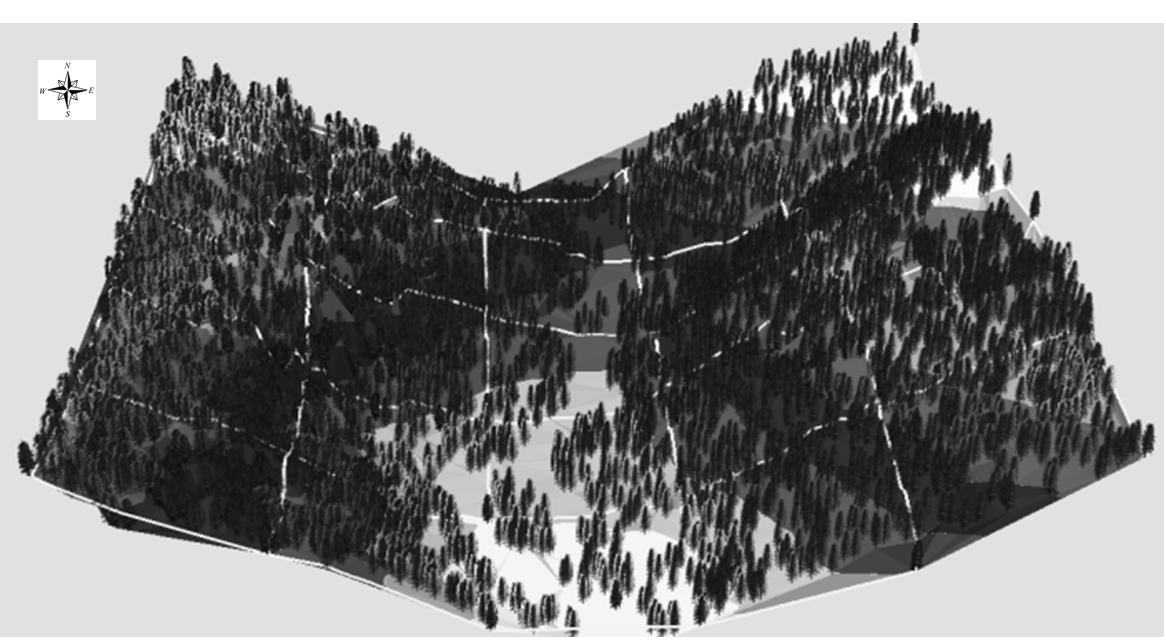


**Table S1**. Descriptive statistics (mean +- 1SE) for environmental variables in each sub-plot. pH: soil acidity, TN: total nitrogen, TP: total phosphorous, NH4: ammonium nitrogen, Soluble N: soluble nitrogen, AP: available phosphorus, AK: available K, CN: ratio of carbon to nitrogen, K: potassium, Ca: calcium and Mg: magnesium

| # | pH | | | Organic C (%) | | | TN g/kg | | | Soluble N g/kg | | | NH4^+–^N g/kg | | | AP mg/kg | | |
| --- | --- | --- | --- | --- | --- | --- | --- | --- | --- | --- | --- | --- | --- | --- | --- | --- | --- | --- |
| 1 | 4.55 | ± | 0.15 | 8.75 | ± | 1.79 | 4.09 | ± | 0.78 | 0.47 | ± | 0.13 | 0.08 | ± | 0.01 | 2.33 | ± | 0.25 |
| 2 | 4.25 | ± | 0.27 | 12.95 | ± | 6.55 | 3.87 | ± | 1.21 | 0.54 | ± | 0.20 | 0.05 | ± | 0.01 | 6.66 | ± | 4.26 |
| 3 | 4.51 | ± | 0.20 | 6.36 | ± | 1.03 | 3.63 | ± | 0.91 | 0.63 | ± | 0.27 | 0.04 | ± | 0.02 | 3.25 | ± | 1.56 |
| 4 | 5.03 | ± | 0.34 | 13.61 | ± | 3.97 | 5.32 | ± | 1.30 | 0.51 | ± | 0.17 | 0.09 | ± | 0.04 | 4.18 | ± | 2.65 |
| 5 | 5.06 | ± | 0.10 | 9.00 | ± | 1.14 | 3.42 | ± | 1.07 | 0.36 | ± | 0.12 | 0.07 | ± | 0.01 | 2.51 | ± | 0.74 |
| 6 | 5.00 | ± | 0.09 | 8.80 | ± | 2.49 | 3.28 | ± | 0.89 | 0.34 | ± | 0.16 | 0.03 | ± | 0.02 | 1.08 | ± | 0.12 |
| 7 | 4.61 | ± | 0.14 | 12.87 | ± | 7.78 | 4.08 | ± | 1.11 | 0.64 | ± | 0.30 | 0.06 | ± | 0.02 | 4.05 | ± | 1.66 |
| 8 | 4.84 | ± | 0.04 | 6.09 | ± | 0.45 | 3.96 | ± | 0.27 | 0.41 | ± | 0.02 | 0.06 | ± | 0.01 | 2.37 | ± | 0.75 |
| 9 | 5.04 | ± | 0.16 | 8.70 | ± | 3.19 | 4.11 | ± | 1.00 | 0.52 | ± | 0.09 | 0.06 | ± | 0.01 | 2.85 | ± | 1.10 |
| 10 | 5.17 | ± | 0.07 | 3.92 | ± | 0.28 | 2.97 | ± | 0.38 | 0.29 | ± | 0.03 | 0.02 | ± | 0.00 | 3.48 | ± | 0.91 |
| 11 | 4.32 | ± | 0.13 | 7.75 | ± | 2.12 | 3.67 | ± | 0.65 | 0.64 | ± | 0.15 | 0.04 | ± | 0.01 | 3.27 | ± | 0.48 |
| 12 | 3.91 | ± | 0.12 | 18.97 | ± | 4.04 | 4.67 | ± | 0.73 | 0.69 | ± | 0.15 | 0.06 | ± | 0.01 | 9.03 | ± | 3.33 |
| 13 | 4.79 | ± | 0.11 | 8.86 | ± | 1.14 | 2.61 | ± | 0.36 | 0.29 | ± | 0.03 | 0.05 | ± | 0.01 | 3.39 | ± | 0.53 |
| 14 | 4.93 | ± | 0.09 | 14.76 | ± | 6.80 | 3.60 | ± | 0.57 | 0.36 | ± | 0.06 | 0.07 | ± | 0.02 | 2.30 | ± | 0.81 |
| 15 | 5.04 | ± | 0.17 | 13.84 | ± | 3.87 | 3.56 | ± | 0.94 | 0.35 | ± | 0.05 | 0.06 | ± | 0.02 | 2.60 | ± | 0.73 |
| 16 | 4.61 | ± | 0.23 | 6.57 | ± | 2.66 | 3.22 | ± | 1.10 | 0.34 | ± | 0.10 | 0.03 | ± | 0.01 | 2.12 | ± | 0.69 |
| 17 | 4.64 | ± | 0.16 | 11.79 | ± | 2.85 | 4.83 | ± | 1.03 | 0.58 | ± | 0.18 | 0.02 | ± | 0.00 | 3.19 | ± | 1.01 |
| 18 | 4.91 | ± | 0.13 | 17.84 | ± | 5.46 | 4.89 | ± | 1.00 | 0.62 | ± | 0.13 | 0.09 | ± | 0.02 | 6.31 | ± | 0.25 |
| 19 | 5.24 | ± | 0.12 | 10.70 | ± | 3.69 | 3.88 | ± | 0.92 | 0.30 | ± | 0.10 | 0.05 | ± | 0.00 | 2.13 | ± | 0.28 |
| 20 | 4.74 | ± | 0.27 | 15.10 | ± | 0.90 | 5.04 | ± | 1.22 | 0.63 | ± | 0.10 | 0.06 | ± | 0.04 | 3.45 | ± | 1.02 |
| 21 | 4.95 | ± | 0.28 | 10.82 | ± | 3.92 | 3.96 | ± | 0.77 | 0.41 | ± | 0.10 | 0.04 | ± | 0.01 | 4.60 | ± | 1.90 |
| 22 | 4.67 | ± | 0.22 | 18.73 | ± | 4.00 | 5.21 | ± | 1.13 | 0.71 | ± | 0.18 | 0.05 | ± | 0.01 | 6.19 | ± | 3.02 |
| 23 | 5.25 | ± | 0.08 | 20.42 | ± | 0.93 | 5.41 | ± | 1.15 | 0.79 | ± | 0.03 | 0.10 | ± | 0.01 | 1.89 | ± | 0.39 |
| 24 | 5.27 | ± | 0.14 | 17.67 | ± | 3.21 | 5.99 | ± | 0.82 | 0.62 | ± | 0.07 | 0.04 | ± | 0.00 | 2.03 | ± | 0.36 |
| 25 | 5.18 | ± | 0.07 | 15.38 | ± | 2.87 | 3.24 | ± | 0.77 | 0.45 | ± | 0.07 | 0.08 | ± | 0.01 | 1.93 | ± | 0.20 |

| # | AK g/kg | | | C/N | | | TP g/kg | | | Mg g/kg | | | K g/kg | | | Ca g/kg | | |
| --- | --- | --- | --- | --- | --- | --- | --- | --- | --- | --- | --- | --- | --- | --- | --- | --- | --- | --- |
| 1 | 0.07 | ± | 0.02 | 12.35 | ± | 0.98 | 0.50 | ± | 0.04 | 7.21 | ± | 0.11 | 18.46 | ± | 0.28 | 7.73 | ± | 0.11 |
| 2 | 0.08 | ± | 0.03 | 17.31 | ± | 3.44 | 0.53 | ± | 0.05 | 7.32 | ± | 0.03 | 18.90 | ± | 0.14 | 7.89 | ± | 0.09 |
| 3 | 0.08 | ± | 0.03 | 10.97 | ± | 2.39 | 0.58 | ± | 0.06 | 7.34 | ± | 0.02 | 19.00 | ± | 0.07 | 7.81 | ± | 0.05 |
| 4 | 0.09 | ± | 0.05 | 14.52 | ± | 0.67 | 0.69 | ± | 0.02 | 7.39 | ± | 0.03 | 19.15 | ± | 0.03 | 7.69 | ± | 0.09 |
| 5 | 0.10 | ± | 0.04 | 17.44 | ± | 4.24 | 0.58 | ± | 0.09 | 7.01 | ± | 0.25 | 18.22 | ± | 0.64 | 6.94 | ± | 0.18 |
| 6 | 0.06 | ± | 0.01 | 16.48 | ± | 4.21 | 0.36 | ± | 0.14 | 7.31 | ± | 0.06 | 18.67 | ± | 0.06 | 7.08 | ± | 0.01 |
| 7 | 0.12 | ± | 0.07 | 15.53 | ± | 5.55 | 0.56 | ± | 0.11 | 7.32 | ± | 0.03 | 18.49 | ± | 0.15 | 7.28 | ± | 0.13 |
| 8 | 0.09 | ± | 0.01 | 9.00 | ± | 0.95 | 0.47 | ± | 0.03 | 7.16 | ± | 0.20 | 18.23 | ± | 0.56 | 7.50 | ± | 0.15 |
| 9 | 0.09 | ± | 0.01 | 12.17 | ± | 2.87 | 0.78 | ± | 0.12 | 7.55 | ± | 0.04 | 19.27 | ± | 0.15 | 7.71 | ± | 0.05 |
| 10 | 0.07 | ± | 0.01 | 7.89 | ± | 0.95 | 0.40 | ± | 0.03 | 7.21 | ± | 0.10 | 19.23 | ± | 0.15 | 7.48 | ± | 0.05 |
| 11 | 0.17 | ± | 0.02 | 12.14 | ± | 2.09 | 0.38 | ± | 0.06 | 7.11 | ± | 0.02 | 18.74 | ± | 0.06 | 7.44 | ± | 0.00 |
| 12 | 0.20 | ± | 0.07 | 23.29 | ± | 1.66 | 0.50 | ± | 0.10 | 6.98 | ± | 0.03 | 18.52 | ± | 0.18 | 7.42 | ± | 0.01 |
| 13 | 0.19 | ± | 0.01 | 20.17 | ± | 2.93 | 0.48 | ± | 0.04 | 7.10 | ± | 0.05 | 19.03 | ± | 0.24 | 7.43 | ± | 0.03 |
| 14 | 0.08 | ± | 0.01 | 21.79 | ± | 6.64 | 0.54 | ± | 0.03 | 7.13 | ± | 0.02 | 19.23 | ± | 0.08 | 7.45 | ± | 0.00 |
| 15 | 0.07 | ± | 0.02 | 23.56 | ± | 6.22 | 0.47 | ± | 0.04 | 7.46 | ± | 0.03 | 19.07 | ± | 0.11 | 8.00 | ± | 0.01 |
| 16 | 0.07 | ± | 0.03 | 11.33 | ± | 0.77 | 0.51 | ± | 0.02 | 7.34 | ± | 0.01 | 18.93 | ± | 0.17 | 7.97 | ± | 0.01 |
| 17 | 0.10 | ± | 0.03 | 14.00 | ± | 0.65 | 0.79 | ± | 0.09 | 7.39 | ± | 0.03 | 18.73 | ± | 0.27 | 7.99 | ± | 0.01 |
| 18 | 0.11 | ± | 0.04 | 20.49 | ± | 2.36 | 0.85 | ± | 0.07 | 7.51 | ± | 0.06 | 18.79 | ± | 0.16 | 8.01 | ± | 0.01 |
| 19 | 0.06 | ± | 0.00 | 15.23 | ± | 1.82 | 0.81 | ± | 0.07 | 7.56 | ± | 0.02 | 19.39 | ± | 0.08 | 8.01 | ± | 0.00 |
| 20 | 0.10 | ± | 0.03 | 19.27 | ± | 3.86 | 0.86 | ± | 0.08 | 7.46 | ± | 0.08 | 19.10 | ± | 0.07 | 7.91 | ± | 0.04 |
| 21 | 0.07 | ± | 0.03 | 14.91 | ± | 2.81 | 0.58 | ± | 0.04 | 7.71 | ± | 0.12 | 19.23 | ± | 0.05 | 7.98 | ± | 0.08 |
| 22 | 0.10 | ± | 0.02 | 20.87 | ± | 0.40 | 0.50 | ± | 0.04 | 7.50 | ± | 0.07 | 18.85 | ± | 0.13 | 7.92 | ± | 0.07 |
| 23 | 0.10 | ± | 0.02 | 23.36 | ± | 3.53 | 0.87 | ± | 0.04 | 7.50 | ± | 0.01 | 19.16 | ± | 0.02 | 7.80 | ± | 0.02 |
| 24 | 0.09 | ± | 0.01 | 16.94 | ± | 0.85 | 0.47 | ± | 0.03 | 7.27 | ± | 0.24 | 18.59 | ± | 0.73 | 7.68 | ± | 0.14 |
| 25 | 0.06 | ± | 0.01 | 28.87 | ± | 3.81 | 0.37 | ± | 0.03 | 7.48 | ± | 0.03 | 19.31 | ± | 0.11 | 7.82 | ± | 0.04 |
